# Effectiveness and Safety of a Fixed-Dose Combination of Dapagliflozin and Vildagliptin in the Treatment of Type 2 Diabetes Mellitus: A Retrospective Study

**DOI:** 10.7759/cureus.97192

**Published:** 2025-11-18

**Authors:** Ami Sanghvi, Sona Warrier

**Affiliations:** 1 Diabetologist, Sanghvi Eye and Diabetes Care Centre, Mumbai, IND; 2 Scientific Services, USV Private Limited, Mumbai, IND

**Keywords:** dapagliflozin, fixed-dose combination, glycemic control, type 2 diabetes mellitus, vildagliptin

## Abstract

Introduction

The fixed-dose combination (FDC) of dapagliflozin 5 mg and vildagliptin 50 mg has been recently approved, but real-world data on its effectiveness and safety are limited. We aimed to evaluate the glycemic efficacy and safety of a combination therapy of dapagliflozin 5 mg and vildagliptin 50 mg in patients with type 2 diabetes mellitus (T2DM) over a three-month period.

Materials and methods

This single-center study retrospectively analyzed data from 49 T2DM patients treated with the FDC of dapagliflozin 5 mg and vildagliptin 50 mg, and complete baseline and three-month follow-up records were analyzed. The primary endpoint was the mean change in glycated hemoglobin (HbA1c). Secondary endpoints included fasting plasma glucose (FPG), postprandial plasma glucose (PPG), lipid profiles, and liver enzymes. Safety assessments included the frequency and nature of adverse events (AEs), such as hypoglycemia, genitourinary infections, gastrointestinal symptoms, and abnormalities in hepatic or renal function. Tolerability was defined as the absence of therapy discontinuation due to adverse effects.

Results

The study enrolled 101 patients with T2DM, of whom 49 patients completed the three-month follow-up (mean age: 55.31 ± 12.04 years) and were included in the analysis. The majority were obese based on Asia-Pacific/Indian body mass index (BMI) criteria (mean BMI: 27.25 ± 4.38 kg/m²). At baseline, the mean HbA1c was 8.87 ± 1.65%, with most patients having HbA1c levels above 8%. After three months of treatment, significant improvements in glycemic parameters were observed, with HbA1c reduced by 1.65 ± 1.23% (p < 0.001), FPG by 68.14 ± 54.31 mg/dL (p < 0.001), and PPG by 122.45 ± 83.08 mg/dL (p < 0.001). Lipid and liver enzyme profiles also showed significant improvement. Subgroup analyses showed consistent glycemic benefits across age, BMI, diabetes duration, and comorbidity categories. The FDC was well tolerated, with no serious AEs and only mild, self-limiting side effects.

Conclusions

This retrospective, single-center, short-term study suggests that dapagliflozin 5 mg and vildagliptin 50 mg may improve glycemic control in patients with T2DM over a three-month period; however, larger, long-term studies are required to confirm durability and safety.

## Introduction

Type 2 diabetes mellitus (T2DM) is complex and driven by several pathophysiological defects, often referred to as the "ominous octet," which includes impaired insulin secretion, insulin resistance, excessive hepatic glucose production, and other metabolic dysfunctions [1]. This intricate interplay of metabolic abnormalities necessitates a treatment approach that targets multiple mechanisms simultaneously rather than relying on monotherapy to achieve sustained glycemic control, particularly in patients with elevated HbA1c at diagnosis [2]. Fixed-dose combinations (FDCs) have therefore emerged as a rational therapeutic strategy, addressing multiple pathways simultaneously, improving metabolic outcomes, reducing pill burden, and enhancing patient adherence [2].

FDCs combining a sodium-glucose cotransporter-2 inhibitor (SGLT-2i) with a dipeptidyl peptidase-4 inhibitor (DPP-4i) have attracted significant clinical interest because they improve glycemic control, reduce fasting and postprandial plasma glucose (PPG), and offer potential benefits on lipid parameters and liver enzymes, while maintaining a low risk of hypoglycemia and weight gain [3,4]. SGLT-2i reduces glucose reabsorption in the proximal renal tubules by inhibiting SGLT-2 proteins, thereby increasing urinary glucose excretion. Currently, four SGLT-2i (canagliflozin, dapagliflozin, empagliflozin, and ertugliflozin) are approved by the U.S. Food and Drug Administration (FDA) for use in adults [5]. On the other hand, DPP-4i prevents the degradation of incretin hormones such as GLP-1 and GIP, enhancing insulin secretion and suppressing glucagon levels in a glucose-dependent manner. This mechanism allows DPP-4i to effectively lower blood glucose levels. Five DPP-4i (sitagliptin, vildagliptin, alogliptin, saxagliptin, and linagliptin) have received regulatory approval. They have since become the second most commonly prescribed class of oral antidiabetic medications after metformin, particularly in patients with chronic kidney disease (CKD) or cardiovascular disease [6]. The combination of SGLT-2i and DPP-4i represents two effective pharmacological strategies that can be integrated with metformin, either as dual therapy or as part of a triple therapy regimen. For patients with T2DM who fail to achieve adequate glycemic control with metformin alone, the combination of DPP-4i and SGLT-2i has demonstrated significant reductions in HbA1c levels [7].

The FDC of vildagliptin and dapagliflozin represents a promising therapeutic approach for managing T2DM, particularly in Asian populations where distinct pathophysiological characteristics, such as a greater degree of β-cell dysfunction, are prevalent [8]. Vildagliptin has been shown to effectively reduce HbA1c levels with a low risk of hypoglycemia and is generally well-tolerated [9]. At a dose of 50 mg, vildagliptin improves glycemic control in patients with T2DM when administered as monotherapy or in combination with other antidiabetic agents. In India, vildagliptin is commonly prescribed due to its ability to reduce the mean amplitude of glycemic excursion, its lower risk of hypoglycemia, and its weight-neutral profile [6]. Dapagliflozin has also demonstrated efficacy in lowering HbA1c levels and promoting weight loss without significantly increasing the risk of hypoglycemia. A 24-week randomized controlled trial reported that dapagliflozin at doses of 1, 2.5, and 5 mg led to significant reductions in HbA1c and body weight in treatment-naïve patients with T2DM [10]. While higher doses of dapagliflozin (10 mg) have been commonly studied, lower doses, such as 5 mg, have also demonstrated efficacy in improving glycemic control with a favorable safety profile [10,11]. A meta-analysis of randomized controlled trials showed that dapagliflozin monotherapy, administered at doses ranging from 1 to 10 mg/day, resulted in significant reductions in HbA1c, fasting plasma glucose (FPG), and body weight compared to placebo. This highlights that even the 5 mg dose was effective in achieving glycemic control and weight reduction [12]. The combination of these two agents is particularly beneficial in Asian populations, where studies have indicated enhanced glycemic improvements with DPP-4i compared to other populations. Vildagliptin therapy has shown robust improvements in glycemic control without increasing the risks of hypoglycemia and weight gain in Asian patients [6,8].

The present study aimed to evaluate the real-world effectiveness and safety of the FDC of dapagliflozin 5 mg and vildagliptin 50 mg in patients with T2DM. The primary objective was to assess the mean change in glycated hemoglobin (HbA1c) from baseline to three months of treatment. The secondary objectives were to evaluate mean changes in FPG, PPG, lipid profile parameters, including total cholesterol (TC), low-density lipoprotein cholesterol (LDL-C), high-density lipoprotein cholesterol (HDL-C), and triglycerides (TG), as well as liver transaminases: alanine transaminase (ALT) and aspartate transaminase (AST). Additionally, the study aimed to assess the safety and tolerability of the treatment, including the occurrence of adverse events (AEs).

## Materials and methods

Study design and ethical considerations

This open-label, single-center, retrospective study was conducted over a three-month period. Data were collected from patient records, focusing on individuals receiving FDC therapy of dapagliflozin 5 mg and vildagliptin 50 mg, as well as those newly diagnosed with T2DM. The study was conducted in accordance with ethical guidelines and regulations, including the National Ethical Guidelines for Conduct of Research Involving Human Participants (ICMR, 2017), the ICH-E6 R3 Guideline (2023) on "Good Clinical Practice," the New Drugs and Clinical Trials Rules (2019), and the Declaration of Helsinki (Taipei, 2016). Before study initiation, approval was obtained from the Institutional Ethics Committee of D Y Patil University School of Medicine (reference number: DYP/IECBH/2025/021, dated January 27, 2025). Given the retrospective nature of the study, a waiver of consent was approved by the IEC; however, written informed consent was obtained from patients wherever possible.

Study population

The study included patients aged 18 years or older, both male and female, diagnosed with T2DM, either newly diagnosed or with uncontrolled diabetes on existing therapies, and having a baseline HbA1c ≥6.0%. The treatment duration for each patient was three months. Patients were included if the FDC (dapagliflozin 5 mg and vildagliptin 50 mg) was prescribed and dispensed within the study period, with documentation of continued use at follow-up. The cohort included both treatment-naïve patients and those who had previously received treatment with other oral agents. Baseline therapy status was not used for stratification in the present analysis. Patients with T1DM on insulin therapy or those with T2DM who had not received the study therapy were excluded.

Of the 101 patients identified at baseline, 49 patients (48.5%) had complete baseline and three-month follow-up data and were included in the final analysis. Patients with incomplete follow-up data were excluded from the efficacy analysis but were reviewed descriptively for safety. Thus, all reported efficacy outcomes are based on the completer cohort (n = 49).

Study endpoints

The primary endpoint of the study was the mean change in HbA1c from baseline to month three. Secondary endpoints included the mean changes in FPG and PPG from baseline to month three, the mean change in lipid profile (including TC, LDL-C, HDL-C, and TG) from baseline to month three, and the mean change in serum transaminases (including AST and ALT) from baseline to month three.

Data collection

Patient records were reviewed to extract demographic, clinical, and biochemical data. Collected demographic parameters included patient age, gender, BMI, diabetic history, comorbid conditions, and concurrent treatments before therapy initiation. Clinical and biochemical parameters collected included baseline and post-treatment blood glucose levels (HbA1c, FPG, PPG), lipid profile (TC, LDL-C, HDL-C, TG), and liver enzymes (ALT, AST). Data on concurrent antidiabetic and lipid-lowering medications were reviewed when available. As this was a retrospective EMR-based analysis, systematic data on medication adjustments or therapy intensifications between baseline and three months were not uniformly recorded.

Safety assessment

AEs were assessed for all 49 patients during the active treatment period and up to two weeks after therapy discontinuation. AE frequencies are expressed as percentages of the 49 patients included in the completer cohort. AEs of interest included genitourinary infections, gastrointestinal symptoms, and hypoglycemia, as defined in patient records. Hypoglycemia was identified as any documented glucose <70 mg/dL or a physician-recorded symptomatic episode.

Statistical analysis

Descriptive statistics were employed to summarize the demographic and baseline characteristics of the study population. Continuous variables, including age (years), weight (kg), and BMI (kg/m²), were expressed as mean ± standard deviation (SD), standard error (SE), minimum (Min), and maximum (Max) values. Categorical variables, such as age groups, HbA1c categories, diabetes duration (years), and BMI classifications, were presented as frequencies and percentages. Unpaired t-tests were used to compare continuous variables between independent groups. The primary endpoint, mean change in HbA1c, was analyzed using paired t-tests for within-group comparisons, depending on the data distribution. Secondary endpoints, including changes in FPG (mg/dL), PPG (mg/dL), lipid profile (mg/dL), and liver enzymes (IU/L), were evaluated using paired t-tests based on the data's normality and the assumption of homogeneity of variances. Subgroup analyses were performed to assess the impact of age (years), BMI (kg/m²), diabetes duration (years), and comorbidities on treatment response, using paired t-tests or ANOVA. A p-value < 0.05 was considered statistically significant for all analyses.

All analyses were conducted using data from patients who completed both baseline and three-month follow-up assessments (completers-only analysis). The analysis excluded patients with missing three-month values rather than imputing data, as the retrospective dataset did not permit reliable estimation of missing outcomes. This approach ensured that all efficacy findings were based on observed data from patients with complete follow-up.

## Results

Baseline characteristics

Of the 101 patients included at baseline, 49 patients (48.5%) completed the three-month follow-up. The remaining 52 patients (51.5%) were excluded due to a lack of documented follow-up visits (n = 28), therapy discontinuation (n = 15), and incomplete laboratory results (n = 9). All results reported here are based on the complete cohort (n = 49).

The mean age of the patients was 55.31 ± 12.04 years. Among them, 65.31% were under 60 years old, and 34.69% were 60 years or older. The majority of patients were obese, with a mean BMI of 26.58 ± 4.42 kg/m². A total of 65.31% of patients had a BMI above 25 kg/m², while 2.04% of patients were underweight. At baseline, the mean HbA1c was 8.87 ± 1.65, with 61.22% of patients presenting with HbA1c levels above 8%. Newly diagnosed diabetes cases comprised 14.29% of the cohort, whereas 24.49% had diabetes for over 10 years. Comorbidities were present in 73.47% of patients, with dyslipidemia being the most common comorbidity. Other comorbidities included hypertension, ischemic heart disease, and hyperthyroidism. Patient demographics and baseline clinical characteristics are summarized in Table 1.

**Table 1:** Patient demographics at baseline (n = 49) Underweight: BMI <18.5 kg/m², normal: BMI 18.5 to 22.9 kg/m², overweight: BMI 23 to 24.9 kg/m², obese: BMI > 25 kg/m² HbA1C: glycated hemoglobin, BMI: basal metabolic rate, SD: standard deviation

Parameter	N = 49
Age (years), mean ± SD	55.31 ± 12.04
Participants ≤60 years, % (n)	73.47 (36)
Participants >60 years, % (n)	26.53 (13)
BMI (kg/m²), mean ± SD	26.58 ± 4.42
Underweight participants, % (n)	2.04 (1)
Normal participants, % (n)	16.33 (8)
Overweight participants, % (n)	16.33 (8)
Obese participants, % (n)	65.30 (32)
HbA1C (%), mean ± SD	8.87 ± 1.65
Participants with HbA1C ≤8%, % (n)	38.78 (19)
Participants with HbA1C >8%, % (n)	61.2 (30)
Duration of diabetes	
<1 year, % (n)	14.29 (7)
1 to <5 year, % (n)	20.41 (10)
5 to <10 years, % (n)	40.81 (20)
≥10 years, % (n)	24.49 (12)
Comorbidity condition	
Absent, % (n)	73.47 (36)
Present, % (n)	26.53 (13)
Hypertension, % (n)	40.82 (20)
Dyslipidemia, % (n)	57.14 (28)
Ischemic heart disease, % (n)	2.04 (1)
Hypothyroid, % (n)	19.23 (10)

Glycemic parameters

After three months of treatment with the FDC of dapagliflozin 5 mg and vildagliptin 50 mg, significant improvements in all glycemic parameters were observed (Table 2).

**Table 2:** Change in glycemic parameters from baseline to three months (n = 49) Data presented as mean ± SD; *p < 0.001 was considered significant for within-group comparison (paired t-test) for baseline vs. three months. FPG: fasting plasma glucose, PPG: postprandial glucose test, HbA1C: glycated hemoglobin, SD: standard deviation

Parameter	At baseline (n = 49)	At 3 months (n = 49)	Change from baseline	p-value
FPG (mg/dL)	190.57 ± 60.56	122.43 ± 26.34	-68.14 ± 54.31	<0.001
PPG (mg/dL)	271.53 ± 93.78	149.08 ± 39.05	-122.45 ± 83.08	<0.001
HbA1C (%)	8.87 ± 1.65	7.22 ± 0.78	-1.65 ± 1.23	<0.001

Mean HbA1c decreased from 8.87 ± 1.65% at baseline to 7.22 ± 0.78% at three months, representing a mean reduction of -1.65 ± 1.23% (p < 0.001). A mean change in FPG from baseline to three months was -68.14 ± 54.31 mg/dL (p < 0.001), while a mean reduction in PPG was -122.45 ± 83.08 mg/dL (p < 0.001). These results showed a substantial and statistically significant improvement in glycemic control over the three-month treatment period.

Laboratory parameters

Changes in lipid profile and liver enzyme parameters are shown in Table 3.

**Table 3:** Change in laboratory parameters from baseline to three months (N = 49) Data presented as mean ± SD; *p < 0.001 was considered significant for within-group comparison (paired t-test) for baseline vs. three months. HDL-C: high-density lipoprotein-cholesterol, LDL-C: low-density lipoprotein-cholesterol, VLDL-C: very low-density lipoprotein-cholesterol, TC: total cholesterol, TG: triglycerides, ALT: alanine transaminase, AST: aspartate transaminase, SD: standard deviation

Parameter	At baseline (n = 49)	At 3 months (n = 49)	Change from baseline	p-value
HDL-C (mg/dl)	44.71 ± 11.49	41.76 ± 9.47	-2.96 ± 6.33	0.002
LDL-C (mg/dl)	101.61 ± 32.06	80.22 ± 29.64	-21.39 ± 23.65	<0.001
VLDL-C (mg/dl)	30.91 ± 16.89	25.35 ± 10.01	-5.56 ± 12.39	0.003
TC (mg/dl)	170.98 ± 36.53	141.96 ± 27.00	-29.02 ± 29.07	<0.001
TG (mg/dl)	154.55 ± 84.43	126.76 ± 50.04	-27.80 ± 61.96	0.003
ALT (U/L)	23.65 ± 11.66	19.61 ± 10.05	-4.04 ± 5.57	<0.001
AST (U/L)	25.24 ± 14.32	20.00 ± 9.43	-5.24 ± 7.91	<0.001

Significant reductions were observed across multiple metabolic markers. TC decreased by 29.02 ± 29.07 mg/dL (p < 0.001), LDL-C by 21.39 ± 23.65 mg/dL (p < 0.001), and VLDL-C by 5.56 ± 12.39 mg/dL (p = 0.003). TG decreased by 27.80 ± 61.96 mg/dL (p = 0.003), while HDL-C also showed a small but statistically significant reduction of 2.96 ± 6.33 mg/dL (p = 0.002). Liver transaminases also improved significantly, with ALT decreasing by 4.04 ± 5.57 U/L (p < 0.001) and AST decreasing by 5.24 ± 7.91 U/L (p < 0.001), suggesting favorable hepatic tolerance to the therapy.

Subgroup analysis

Subgroup analyses indicated consistent glycemic improvements across different patient subgroups (Table 4).

**Table 4:** Subgroup analysis of glycemic parameters for change from baseline to three months (n = 49) Data presented as mean ± SD; *p < 0.001 was considered significant for within-group comparison for baseline vs. three months (paired t-test). Underweight: BMI <18.5 kg/m², normal: BMI 18.5 to 22.9 kg/m², overweight: BMI 23 to 24.9 kg/m², obese: BMI > 25kg/m². BMI classification was based on the Asia-Pacific/Indian consensus guidelines, where overweight is defined as BMI ≥23 kg/m² and obesity as BMI ≥25 kg/m². FPG: fasting plasma glucose, PPG: postprandial glucose test, HbA1C: glycated hemoglobin, BMI: body mass index, SD: standard deviation

Parameters		Baseline (n = 49)		3 months (n = 49)		Change at 3 months		*p-value
		n	Mean ± SD	n	Mean ± SD	n	Mean ± SD	
Age subgroups								
FPG	<60 yrs.	32	194.31 ± 55.34	32	124.31 ± 27.31	32	-70.00 ± 47.29	<0.001
	>60 yrs.	17	183.53 ± 70.63	17	118.88 ± 24.82	17	-64.65 ± 67.05	0.001
PPG	<60 yrs.	32	285.06 ± 91.79	32	155.03 ± 36.14	32	-130.03 ± 85.84	<0.001
	>60 yrs.	17	246.06 ± 94.89	17	137.88 ± 42.88	17	-108.18 ± 78.10	<0.001
HbA1C	<60 yrs.	32	8.97 ± 1.75	32	7.24 ± 0.90	32	-1.73 ± 1.30	<0.001
	>60 yrs.	17	8.68 ± 1.48	17	7.18 ± 0.49	17	-1.49 ± 1.11	<0.001
BMI subgroups								
FPG	Underweight	1	221.00 ± 0	1	126.00 ± 0	1	-95.00 ± 0	-
	Normal weight	8	179.75 ± 48.22	8	118.00 ± 26.32	8	-61.75 ± 50.37	0.001
	Overweight	8	172.63 ± 28.67	8	121.00 ± 18.84	8	-51.63 ± 26.02	<0.001
	Obesity	32	196.81 ± 69.18	32	123.78 ± 28.81	32	-73.03 ± 60.93	<0.001
PPG	Underweight	1	178.00 ± 0	1	109.00 ± 0	1	-69.00 ± 0	-
	Normal weight	8	262.75 ± 87.38	8	167.75 ± 33.45	8	-95.00 ± 61.49	0.003
	Overweight	8	263.00 ± 92.76	8	138.25 ± 21.54	8	-124.75 ± 89.64	0.006
	Obesity	32	278.78 ± 97.85	32	148.38 ± 42.79	32	-130.41 ± 87.56	<0.001
HbA1C	Underweight	1	10.80 ± 0	1	7.40 ± 0	1	-3.40 ± 0	-
	Normal weight	8	8.51 ± 1.21	8	6.95 ± 0.51	8	-1.56 ± 0.95	0.002
	Overweight	8	8.43 ± 2.09	8	7.01 ± 0.82	8	-1.41 ± 1.31	0.018
	Obesity	32	9.01 ± 1.64	32	7.33 ± 0.83	32	-1.67 ± 1.29	<0.001
Duration of DM								
FPG	<1 yrs	7	251.00 ± 57.00	7	120.43 ± 17.75	7	-130.57 ± 54.33	<0.001
	1 to <5 year	10	188.00 ± 86.99	10	115.10 ± 14.65	10	-72.90 ± 85.56	0.025
	5 to <10 years	20	181.75 ± 44.83	20	127.75 ± 32.21	20	-54.00 ± 27.81	<0.001
	≥10 years5	12	172.17 ± 41.59	12	120.83 ± 27.97	12	-51.33 ± 27.61	<0.001
PPG	<1 yrs	7	356.43 ± 109.25	7	156.86 ± 50.02	7	-199.57 ± 113.87	0.004
	1 to <5 year	10	263.20 ± 85.64	10	150.90 ± 38.55	10	-112.30 ± 76.53	0.001
	5 to <10 years	20	269.60 ± 77.18	20	152.70 ± 33.02	20	-116.90 ± 69.89	<0.001
	≥10 years5	12	232.17 ± 96.07	12	137.00 ± 44.33	12	-95.17 ± 71.24	<0.001
HbA1C	<1 yrs	7	9.19 ± 1.09	7	6.89 ± 0.34	7	-2.30 ± 1.25	0.003
	1 to <5 year	10	8.25 ± 0.84	10	7.23 ± 0.45	10	-1.02 ± 0.61	<0.001
	5 to <10 years	20	9.22 ± 2.19	20	7.37 ± 1.07	20	-1.86 ± 1.48	<0.001
	≥10 years	12	8.61 ± 1.29	12	7.17 ± 0.59	12	-1.44 ± 0.97	<0.001
Comorbidity								
FPG	Present	36	173.94 ± 50.33	36	121.64 ± 25.64	36	-52.31 ± 39.77	<0.001
	Absent	13	236.62 ± 64.47	13	124.62 ± 29.17	13	-112.00 ± 65.94	<0.001
PPG	Present	36	250.72 ± 87.99	36	147.28 ± 38.74	36	-103.44 ± 73.24	<0.001
	Absent	13	329.15 ± 87.76	13	154.08 ± 41.04	13	-175.08 ± 88.78	<0.001
HbA1C	Present	36	8.71 ± 1.64	36	7.15 ± 0.76	36	-1.56 ± 1.27	<0.001
	Absent	13	9.30 ± 1.66	13	7.41 ± 0.84	13	-1.89 ± 1.14	<0.001

Patients aged below 60 years achieved a mean HbA1c reduction of -1.73 ± 1.30% (p < 0.001), while those aged 60 years or older showed a reduction of -1.49 ± 1.11% (p < 0.001), with comparable declines in fasting and PPG levels in both groups. When analyzed by BMI, the most significant reduction in HbA1c was observed among obese patients (-1.67 ± 1.29%, p < 0.001), although all BMI categories demonstrated statistically significant improvements in glycemic parameters. Regarding diabetes duration, patients with less than one year of diabetes exhibited the most significant decrease in HbA1c (-2.30 ± 1.25%, p = 0.003). In contrast, those with a disease duration of ten years or more achieved a reduction of -1.44 ± 0.97% (p < 0.001). Similarly, patients without comorbidities experienced a slightly greater improvement in HbA1c (-1.89 ± 1.14%, p < 0.001) compared with those having comorbid conditions (-1.56 ± 1.27%, p < 0.001). Overall, these findings indicate that the FDC of dapagliflozin 5 mg and vildagliptin 50 mg demonstrated consistent glycemic efficacy across diverse subgroups, including older adults, obese individuals, and patients with varying disease durations or comorbidity burdens.

Safety and tolerability

The combination therapy demonstrates a favorable safety profile. Mild AEs were reported in a small proportion of patients (gastrointestinal discomfort, 2%; genitourinary infection, 1.5%; and mild hypoglycemia, 1.5%), calculated based on 49 exposed patients. No severe AEs, hepatic enzyme elevations, or renal complications were observed during treatment or within two weeks post-treatment.

## Discussion

This study evaluated the real-world effectiveness and safety of the FDC of dapagliflozin 5 mg and vildagliptin 50 mg in patients with T2DM using a completer-only analysis. Among patients who completed three months of follow-up, the combination produced substantial and statistically significant reductions in HbA1c, FPG, and PPG, together with favorable changes in lipid and liver enzyme profiles. These findings are significant in light of the rising prevalence of diabetes in regions such as India, where more than half of the patients fail to achieve target glycemic control with conventional therapies [13].

The FDC of dapagliflozin 5 mg and vildagliptin 50 mg received regulatory approval from the Drug Controller General of India in February 2023 for the management of T2DM [14]. This approval highlighted the therapeutic potential of this combination, particularly for patients who may not tolerate higher doses. Beyond glycemic control, expert opinion from India suggests that healthcare providers prefer this FDC for obese patients with T2DM [2]. Traditional FDCs often include metformin alongside another agent, but they can be associated with side effects such as gastrointestinal discomfort, weight gain, hypoglycemia, and fluid retention. In contrast, DPP-4i and SGLT-2i have a more favorable safety profile and a lower risk of these complications. Additionally, SGLT-2i, alone or in combination with DPP-4i, has demonstrated clinically significant weight reduction [2,14]. The selection of vildagliptin 50 mg instead of 100 mg is based on evidence that this dose achieves approximately 80% DPP-4 receptor inhibition for 12 hours. This inhibition is sufficient for effective glycemic control while reducing the risk of adverse effects, such as hepatic enzyme elevation [14].

This study indicated significant improvements in glycemic parameters. The mean HbA1c levels significantly decreased by 1.65%. Additionally, FPG and PPG levels showed notable reductions of 68.14 mg/dL and 122.45 mg/dL, respectively. These findings highlight the strong glucose-lowering potential of this combination. Prior research has shown similar additive effects when these classes of drugs are used together [15].

Subgroup analyses revealed that the combination therapy is effective across various populations. In our study, older patients, obese individuals, and those with a shorter duration of diabetes (less than one year) all benefited significantly. The larger HbA1c reduction in patients with a shorter diabetes duration suggests that early intervention might help preserve β-cell function and yield better long-term control [16]. This finding is important for tailoring treatment strategies to individual patient needs.

The study's data confirmed the favorable safety profile of the dapagliflozin 5 mg and vildagliptin 50 mg combination therapy. This supports the suitability of this regimen for a broad patient population, particularly those at risk of drug-related complications. These results are in line with the established safety profiles of dapagliflozin and vildagliptin, which have been well-documented in previous studies [6,17].

The 2025 American Diabetes Association guidelines emphasize individualized therapy, particularly for patients with comorbidities such as obesity, cardiovascular disease, and CKD. The guidelines recommend SGLT-2i for their proven cardiovascular and renal benefits and DPP-4i for their glucose-lowering efficacy with minimal risk of hypoglycemia [18]. The combination of dapagliflozin and vildagliptin aligns with these recommendations, making it a suitable option for newly diagnosed patients and those with specific comorbidities. The selection of dapagliflozin 5 mg and vildagliptin 50 mg also offers several advantages. SGLT-2i, such as dapagliflozin, promote glucosuria, which triggers an increase in endogenous glucose production, partially offsetting their glucose-lowering effect [7]. The ability of vildagliptin 50 mg to block 80% of DPP-4 receptors is sufficient for substantial glycemic control without requiring higher doses that might increase AEs [14]. This dosing strategy is particularly relevant for elderly or frail patients who may not tolerate higher drug exposures and is also approved to be used in this patient population.

While our findings are promising, the study has several limitations. Given the retrospective design and high rate of incomplete follow-up, the efficacy analysis was restricted to patients with complete baseline and three-month data. This completer-only approach ensured that results reflected observed, verified clinical outcomes without relying on statistical imputation. Although this may introduce some selection bias, it provides a reliable and realistic depiction of treatment effectiveness among patients who continued therapy in routine practice. The study’s retrospective, single-center nature may introduce inherent biases, and data accuracy depended on the availability of medical records, which may not have captured all relevant clinical variables. The relatively short follow-up duration of three months limits the assessment of long-term outcomes such as the durability of glycemic control, β-cell preservation, and prevention of microvascular or macrovascular complications.

Furthermore, while prior literature has established cardiovascular and renal benefits of SGLT-2i, the current study was not designed to evaluate these endpoints. Therefore, the findings should be interpreted as short-term, real-world evidence of improved glycemic and metabolic parameters with dapagliflozin and vildagliptin FDC therapy, rather than definitive proof of long-term disease modification. Nevertheless, the results add valuable early evidence from routine clinical settings in India, and future prospective, multicenter studies with longer follow-up are warranted to confirm the long-term benefits, durability, and safety of this therapeutic approach.

## Conclusions

This retrospective, real-world study suggests that the FDC of dapagliflozin 5 mg and vildagliptin 50 mg significantly improves short-term glycemic control and metabolic parameters in individuals with T2DM. The treatment was effective across various patient subgroups with a favorable safety profile. Although the completer-only design may overrepresent patients more adherent to therapy, it provides a realistic depiction of treatment outcomes in routine clinical practice. Given the study's retrospective design, high attrition rate, and short follow-up period, the results should be interpreted with caution. Larger, prospective, multicenter studies with longer follow-up durations are warranted to confirm these findings and further assess the long-term safety, durability of glycemic control, and cardiometabolic benefits of this combination therapy.
